# Interspecific variation and environmental drivers of rhizosphere microbiomes in endemic *Impatiens* species

**DOI:** 10.3389/fpls.2025.1567041

**Published:** 2025-05-08

**Authors:** Zhansheng Tang, Lina Xie, Junjie Chen, Yunquan Wang, Yalei Li

**Affiliations:** ^1^ College of Ecology, Lishui University, Lishui, Zhejiang, China; ^2^ The Administration Center of Jiulong Mountain National Nature Reserve, Lishui, Zhejiang, China; ^3^ College of Life Sciences, Zhejiang Normal University, Jinhua, Zhejiang, China

**Keywords:** plant-soil interactions, microbial diversity, endemic species, environmental filtering, elevation gradient

## Abstract

**Introduction:**

Understanding rhizosphere microbiomes of endemic plants is crucial for their conservation, yet it remains poorly explored, in particular for species-rich genera with high endemism rates like *Impatiens*.

**Methods:**

We investigated rhizosphere bacterial and fungal communities of five *Impatiens* species (including two endemics) across altitude gradients in subtropical China using high-throughput sequencing. We analyzed microbial community structure in relation to environmental factors, soil properties, and plant traits.

**Results:**

Significant interspecific variations were observed in both bacterial and fungal communities, with endemic species harboring distinct microbiomes. Fungal communities showed stronger species-specificity than bacterial communities, particularly in the endemic *I. suichangensis*. Redundancy analysis revealed that elevation explained a substantially higher proportion of fungal community variation compared to bacterial variation. Soil nutrients and pH strongly influenced microbial community structure, while plant traits showed species-specific correlations with particular microbial taxa. Notably, companion plant diversity positively correlated with fungal diversity indices.

**Discussion:**

These findings highlight the complex associations among plant traits, environmental factors, and rhizosphere microbiomes in *Impatiens* species, providing correlative evidence for potential plant-microbe interactions in endemic plant species. Our results emphasize the importance of considering both above- and below-ground components in conservation strategies for endemic plant species.

## 1 Introduction

Plant-microbiome interactions are now recognized as fundamental drivers of plant adaptation, community assembly, and ecosystem functioning (Philippot et al., 2013; Fitzpatrick et al., 2020). While agricultural systems have dominated rhizosphere microbiome research, critical knowledge gaps exist for endemic plants in threatened biodiversity hotspots (de Faria et al., 2021; Pantigoso et al., 2022). This deficiency is particularly evident in diverse plant genera with high endemism rates, where understanding belowground interactions could inform essential conservation strategies. The genus *Impatiens* L. (Balsaminaceae) presents an exceptional model system for investigating these questions, owing to its extraordinary evolutionary radiation, high endemicity, and vulnerability to environmental changes (Yu et al., 2016).

With over 1,000 documented species distributed globally, *Impatiens* spans tropical, subtropical, and warm temperate mountainous regions throughout the Old World (Chen et al., 2008; Janssens et al., 2009). Beyond their ornamental value, *Impatiens* species hold significant medicinal importance due to their distinctive secondary metabolites, which potentially influence rhizosphere microbial communities through specific root exudates (Yang et al., 2001; Gaggini et al., 2018). China constitutes a major center of *Impatiens* diversity, hosting 352 species, including 273 endemic taxa as of 2022 (Yuan et al., 2022). However, these species—particularly those with restricted distributions—face increasing threats from environmental changes and anthropogenic disturbances (Zhang et al., 2015; Yu et al., 2019; The IUCN, 2022). Despite their ecological and conservation significance, research on rhizosphere microbiomes of wild *Impatiens* species remains notably scarce, creating a substantial knowledge gap regarding how these diverse plant species interact with soil microbial communities. This research gap is especially concerning for endemic *Impatiens* species with extremely restricted distributions, as they may have evolved specialized microbial associations vital for their persistence under changing environmental conditions.

Recent advances in plant-soil interaction research have underscored the crucial role of rhizosphere microorganisms in sustaining biodiversity and plant health (Bardgett and van der Putten, 2014; Prober et al., 2015; Li et al., 2024). Plants actively recruit beneficial microorganisms through specialized root exudates, which subsequently enhance plant growth, bolster stress resistance, and modulate plant community coexistence (Bulgarelli et al., 2013; van der Heijden et al., 2016). These plant-microbe interactions are especially significant in endemic-rich plant assemblages, where rhizosphere microbes likely facilitate local adaptation and niche differentiation (Fitzpatrick et al., 2018). Although rhizosphere microbial communities are known to be shaped by soil physicochemical properties (Dini-Andreote et al., 2015; Tripathi et al., 2018), plant characteristics (Gao et al., 2015), and vegetation composition (Chen et al., 2017), the mechanisms driving species-specific differences in rhizosphere microbiomes remain poorly elucidated, particularly for endemic-rich genera such as *Impatiens*. Several fundamental questions persist: Do endemic plant species harbor distinct rhizosphere communities compared to widespread congeners? How do environmental gradients influence these plant-microbe associations? To what extent do plant traits versus environmental factors drive rhizosphere community assembly? Addressing these questions extends beyond academic curiosity—it has profound implications for the conservation of endemic plant species confronting global environmental change.

The Jiulong Mountain National Nature Reserve in southwestern Zhejiang Province constitutes an ideal natural laboratory for investigating plant-microbe interactions among naturally co-occurring congeners with contrasting distribution patterns. The reserve hosts five *Impatiens* species: *I. suichangensis* (*I*. sui), *I. chloroxantha* (*I.* chl), *I. tienmushanica* lon (*I*. tie. lon), *I. jiulongshanica* (*I*. jiu), and *I. chekiangensis* (*I*. che), with *I*. sui and *I*. tie. lon being endemic to this reserve and Zhejiang Province, respectively. Notably, *I. sui* has been classified as Near Threatened (NT) in the International Union for Conservation of Nature (IUCN) Red List of Threatened Species. We hypothesized that: (1) endemic *Impatiens* species harbor distinct rhizosphere microbial communities compared to their widespread congeners; (2) environmental factors exert stronger influences on fungal communities than bacterial communities along elevation gradients; and (3) plant traits and companion species diversity significantly influence rhizosphere microbial community composition. To test these hypotheses, we measured plant growth parameters, companion species diversity, soil physicochemical properties, and environmental characteristics, while simultaneously analyzing bacterial and fungal community structure and diversity. Our findings provide novel insights into plant-soil interactions in endemic taxa and establish a scientific foundation for the conservation of wild *Impatiens* resources in subtropical regions.

Our comprehensive investigation of rhizosphere microbiomes across five *Impatiens* species with distinct distribution patterns offers an unprecedented opportunity to address these fundamental ecological questions. The findings from this study carry significant implications for: (1) elucidating the ecological and evolutionary processes that shape plant-microbe interactions in diverse plant genera; (2) developing effective conservation strategies for endemic plant species that integrate both above- and below-ground components; and (3) advancing our understanding of microbial community assembly along environmental gradients in subtropical forest ecosystems. By characterizing the intricate associations among plant traits, environmental factors, and microbial communities, this research establishes critical baseline data essential for monitoring and preserving the unique plant-microbe relationships that may underpin the persistence of these ecologically valuable and threatened plant resources.

## 2 Materials and methods

### 2.1 Study site description

This study was conducted in the Jiulong Mountain National Nature Reserve (28°19′10″-28°24′43″N, 118°49′38″-118°55′03″E), Zhejiang Province, China. Located in southwestern Suichang County at the junction of Zhejiang, Fujian, and Jiangxi provinces, the reserve sits on a branch of the Xianxialing Mountains within the Wuyi Mountain system. The region exhibits a mid-subtropical humid monsoon climate with well-defined seasonal patterns. The mean annual temperature is approximately 17°C, with annual precipitation of 1,855 mm and mean annual relative humidity of 83%.

The reserve’s complex topography creates distinct vertical climate zones, with higher elevations displaying near-temperate climatic characteristics. Aluminum-rich soils predominate throughout the area, with an elevational gradient of soil types including mature red soils, red soils, yellow-red soils, and red-yellow soils. The vegetation consists primarily of subtropical evergreen broad-leaved forest, representing one of East China’s best-preserved areas of primary vegetation (Liu et al., 2024). The plant community exhibits well-developed stratification and rich species diversity, creating suitable habitats for various *Impatiens* species.

### 2.2 Survey methods

#### 2.2.1 Survey of *Impatiens* resource distribution

Field surveys were conducted during the peak growing season of *Impatiens* (July-September 2023) using combined transect and quadrat methods along an elevational gradient (380–1050 m). GPS coordinates and elevation were recorded for each sampling site, along with environmental parameters. For each 1 m × 1 m quadrat, we visually estimated rock exposure (percentage of exposed rock surface area) and litter cover (percentage of ground covered by litter). Litter layer thickness was measured to the nearest 0.1 cm by averaging values from five randomly selected points per quadrat. Humus layer thickness was determined to the nearest 0.1 cm by averaging measurements from three randomly selected points per quadrat through soil excavation.

#### 2.2.2 Environmental factors and companion species survey

We established 4 to 15 quadrats (1 m × 1 m) for each *Impatiens* species (**Table 1**). A consistent set of environmental factors, plant traits, soil properties, and companion plant diversity measurements were recorded for each quadrat (detailed in **Supplementary Table S1**). Standard methods were used to measure topographical parameters (slope gradient, aspect, and position), environmental characteristics, and soil properties at each site. All vascular plants within each quadrat were identified according to Flora of China and recorded, ensuring only one *Impatiens* species was present per quadrat. To assess companion plant community diversity, we calculated three diversity indices from the companion plant data:

**Table 1:** Survey of five *Impatiens* species in Jiulong Mountain: plot details and habitat characteristics.

Species	Number of quadrats	Frequency	Number of *Impatiens* individuals	Altitude/m	Soil type	Main companion plant species
*I*. sui	6	15.80%	41	1020-1050	Umbrisols	*C. lanceolata* *Emmenopterys henryi* *Cornus officinalis* var. *huaxiensis*
*I*. chl	6	15.80%	51	450-850	Acrisols, Umbrisols	*C. lanceolata* *Alnus cremastogyne* *Cyclobalanopsis glauca*
*I*. tie. lon	4	10.50%	25	980-1000	Umbrisols	*C. lanceolata* *Choerospondias axillaris*, *Hydrangea chungii*
*I*. jiu	7	18.40%	42	750-965	Umbrisols	*C. lanceolata* *Cryptomeria japonica* *Liquidambar formosana*
*I*. che	15	39.40%	114	380-1000	Acrisols, Ferralsols, Arenosols, Cambisols	*C. lanceolata* *Alnus cremastogyne* *Pterostyrax corymbosus*

The Shannon-Wiener diversity index (H'), Simpson diversity index (D), and Pielou evenness index (J) were calculated as follows (Equations 1–3),


(1) H'=−∑i=1S(Pi×lnPi)

(2) $$\text{D}=1-\sum_{\text{i}=1}^{\text{S}}{\text{P}}_{\text{i}}^{2}$$

(3) $$\text{J}={\text{H}}^{\prime }/\text{ln}(\text{S})$$

where P_i_ is the relative abundance of species i, and S is the total number of species.

#### 2.2.3 Measurement of basic biological traits

We systematically sampled *Impatiens* plants within each quadrat following a consistent protocol. When a quadrat contained more than 8 *Impatiens* individuals, we randomly selected 8 plants; otherwise, all individuals were sampled. This approach yielded an average of 7 plants per quadrat, with 25–114 individuals sampled per species across all quadrats (**Table 1**). For each selected plant, we recorded several morphological traits: crown diameter along both long (d_1_) and short (d_2_) axes, plant height, branch diameter, number of leaves, and leaf area. Crown diameter was measured to the nearest 0.1 cm by averaging projection diameters along east-west and north-south directions using a tape measure. Plant height was measured as the vertical distance from ground level to the highest point of the plant. Branch diameter was measured to the nearest 0.01 cm using a vernier caliper at 2 cm above ground level. We counted all intact leaves per plant and determined average leaf area by measuring three randomly selected, fully expanded mature leaves per plant using a portable leaf area meter. Crown area (Equation 4) and total leaf area (Equation 5) were calculated as follows,


(4) $$\text{Crown}=\frac{1}{4}\times \text{π}\times {\text{d}}_{1}\times {\text{d}}_{2}$$

(5) Leaf area=Blade number×average leaf area

#### 2.2.4 Determination of soil physicochemical properties

For each quadrat, we collected soil samples from the 0–20 cm layer at three randomly selected points after removing surface debris. These samples were combined, thoroughly mixed to create a composite sample, and air-dried in the laboratory. Available potassium (AK) was determined using 1N ammonium acetate (NH_4_OAC) extraction followed by flame photometry. Available nitrogen (AN) was measured using the alkali-diffusion method. Available phosphorus (AP) was analyzed using the Olsen method (0.5 mol/L NaHCO_3_ extraction followed by molybdenum-antimony colorimetric determination). Soil organic carbon (SOC) was determined using the potassium dichromate oxidation method with external heating. Soil pH was measured using a potentiometric method (soil:water ratio = 1:2.5). Soil moisture content was determined by oven-drying at 105°C to constant weight. Soil temperature was measured at 10 cm depth using a soil thermometer.

#### 2.2.5 Collection of rhizosphere soil samples and DNA extraction

Three healthy individuals of each *Impatiens* species (showing no signs of disease or stress, with intact root systems) were randomly selected at each sampling quadrat for rhizosphere soil collection. After carefully excavating the root system to a depth of 20 cm using sterile tools, bulk soil was removed by gently shaking the roots. Rhizosphere soil (defined as soil within 0–2 mm from the root surface) was collected using a sterilized soft-bristled brush. For each plant, approximately 5 g of rhizosphere soil was collected. For each plant, approximately 5 g of rhizosphere soil was collected. The collected soil samples from 3 plants for the same species at each sampling quadrat were thoroughly homogenized to create a composite sample, immediately placed in sterile 10 mL centrifuge tubes, and transported to the laboratory and stored at -70°C.

Total soil DNA was extracted from 0.5 g of each composite sample using the FastDNA^®^ SPIN Kit for Soil (MP Biomedicals, USA) following the manufacturer’s protocol, with minor modifications to optimize DNA yield: the bead-beating step was performed twice at 6.0 m/s for 40 s using a FastPrep-24™ instrument. DNA concentration and purity were measured using a NanoDrop 2000 spectrophotometer (Thermo Scientific, USA), with acceptable quality thresholds set at: concentration ≥50 ng/μL, A260/A280 ratio between 1.8-2.0, and A260/A230 ratio >1.7. DNA integrity was assessed by 1% agarose gel electrophoresis run at 120V for 30 minutes. Only DNA samples showing clear bands without significant degradation were used for subsequent analyses. Qualified samples were then normalized to 20 ng/μL and submitted for high-throughput sequencing analysis targeting the V3-V4 regions of bacterial 16S rRNA and the ITS1 region for fungi. The primer sequences are listed in detail in **Table 2**.

**Table 2:** Primer sequences.

Primers	Primer Type	F-End Sequence	R-End Sequence
338F_806	Bacteria 16S rRNA	ACTCCTACGGGAGGCAGCAG	GGACTACHVGGGTWTCTAAT
ITS1F_ITS2R	Fungus ITS	CTTGGTCATTTAGAGGAAGTAA	GCTGCGTTCTTCATCGATGC

### 2.3 Data analysis

#### 2.3.1 Bioinformatic analysis

Amplicon sequencing was performed on the Illumina MiSeq platform (Majorbio Biotechnology Co. Ltd, Shanghai, China) using 2 × 300 bp paired-end sequencing. Raw sequencing data were processed using QIIME2 (version 2023.7). Initial quality filtering removed sequences with average quality scores <25, ambiguous bases, and primer mismatches. The DADA2 plugin was used for denoising with optimized parameters for both 16S rRNA and ITS sequences, with trimming and truncation lengths adjusted based on sequence quality profiles. Chimeric sequences were removed using the consensus method in DADA2.

High-quality sequences were clustered into Amplicon Sequence Variants (ASVs) at 97% similarity using the vsearch plugin. Taxonomic annotation was performed using the q2-feature-classifier with the Silva database (Release 138.1) for 16S rRNA sequences and the UNITE database (Version 8.3) for ITS sequences, using a confidence threshold of 0.7. To ensure comparability among samples, the sequencing depth was normalized by rarefying to 30,000 sequences per sample for bacteria and 20,000 sequences per sample for fungi. These thresholds represented the minimum sequence counts across our sample set while maintaining adequate coverage (Good’s coverage >0.97). Any samples failing to meet these thresholds were excluded from subsequent analyses.

#### 2.3.2 Statistical analysis

Statistical analyses were performed using SPSS software (version 19.0). Data were first tested for normality using the Shapiro-Wilk test. For normally distributed data, one-way ANOVA followed by Tukey’s HSD test was used to compare plant traits, soil physicochemical properties, and microbial diversity indices among different *Impatiens* species. For non-normally distributed data or data with unequal variances, Tamhane’s T2 *post-hoc* test was applied. The significance level was set at *p* < 0.05 for all statistical analyses. Microbial α-diversity indices (ACE, Chao1, Simpson diversity, Shannon-Wiener diversity) were calculated using the vegan package in R. To assess differences in microbial community composition among *Impatiens* species, Non-metric Multidimensional Scaling (NMDS) based on Bray-Curtis distances was employed, with significance tested using Analysis of Similarities (ANOSIM).

#### 2.3.3 Community composition and environmental correlation analysis

Venn diagrams were used to visualize shared and unique ASVs among different *Impatiens* species. Stacked bar charts were created to display microbial community composition at phylum and genus levels. Mantel tests were performed to analyze correlations between environmental factors and microbial community composition. Redundancy Analysis (RDA) was conducted to investigate the influence of environmental factors on microbial community composition, with the significance of RDA axes assessed using Monte Carlo permutation tests (999 permutations). The environmental factors included plant traits (crown, plant height, branch diameter, blade number, leaf area), soil physicochemical properties (AK, AN, AP, SOC, pH, soil humidity, soil temperature), and environmental characteristics (rock exposure, litter layer, litter cover, humus layer). After collinear variables were removed, Spearman correlation analysis was used to evaluate relationships between environmental factors and microbial α-diversity indices.

#### 2.3.4 Data visualization

Figures were created using the ggplot2 package in R (version 4.1.0), with all numerical data presented as mean ± standard deviation (Mean ± SD). For visualizing differences between species, error bars representing standard deviation were included on bar charts. Statistical significance (*p* < 0.05) between groups was indicated using different letters above bars. Correlation heatmaps were generated using the corrplot package, with color intensity representing Spearman correlation coefficient values and asterisks indicating significance levels. For ordination plots (NMDS), 95% confidence ellipses were calculated and displayed to visualize group clustering, with stress values reported to indicate goodness of fit. RDA triplots were constructed to simultaneously represent samples, environmental variables, and major microbial taxa, with arrow length proportional to the explanatory power of each environmental factor.

#### 2.3.5 Taxonomic resolution selection

For the analysis of rhizosphere microbial communities, we focused on genus-level taxonomic resolution in the main text while including ASV-level analyses in **Supplementary Materials**. We chose genus-level analysis as our main approach for several compelling biological and technical reasons.

First, genus-level analysis provides a more functional perspective on microbial communities, as many ecological functions are conserved at the genus level (Philippot et al., 2010; Martiny et al., 2015). Second, genus-level analysis reduces the inherent noise in microbial community data, enabling more robust ecological interpretations that are less influenced by rare taxa (Shade et al., 2014). Third, our preliminary comparison between ASV and genus-level analyses revealed consistent community differentiation patterns across *Impatiens* species at both taxonomic levels (**Supplementary Figure S1**), indicating that the key ecological signals in our dataset are preserved at the genus level.

Additionally, genus-level analysis facilitates more direct comparisons with existing literature on plant-associated microbiomes, as many studies report findings at this taxonomic resolution. While ASV-level analysis provides higher resolution for detecting subtle community differences, the genus-level approach provides a more biologically interpretable framework for understanding ecological and functional patterns in rhizosphere microbial communities of *Impatiens* species.

## 3 Results

### 3.1 *Impatiens* species exhibit distinct elevational distribution patterns in Jiulong Mountain

We investigated the germplasm resources and habitat characteristics of five *Impatiens* species (*I*. sui, *I.* chl, *I*. tie. lon, *I*. jiu, and *I*. che) distributed in the Jiulong Mountain Nature Reserve (**Table 1**). Across 38 established quadrats, these *Impatiens* species showed distinct elevational differentiation: *I*. chl primarily occurred at low elevations (450–850 m), *I*. jiu at mid-to-high elevations (750–965 m), *I*. sui and *I*. tie. lon were restricted to high elevations (980–1050 m), while *I*. che exhibited widest elevational range (380–1000 m).

Regarding soil types, *I*. sui, *I*. jiu, and *I*. tie. lon were mainly found in areas with black soils characterized by high organic matter content, whereas *I*. chl and *I*. che showed adaptation to multiple soil types including red-yellow soils, yellow soils, and sandy soils. Although Chinese fir (*Cunninghamia lanceolata*) dominated all *Impatiens* distribution areas, the composition of companion tree species varied with elevation. These distinct distribution patterns suggest that different *Impatiens* species are associated with specific environmental conditions, which may correspond to differences in their rhizosphere microbial communities.

### 3.2 Endemic *I*. tie. lon displays significantly larger crown width and plant height among five Impatiens species

Measurement and comparison of primary growth parameters among the five *Impatiens* species revealed significant interspecific differences (**Table 3**). For crown width, *I*. tie. lon was significantly larger than other species, while *I*. sui, *I*. chl, and *I*. che exhibited relatively smaller crown sizes. Plant height measurements showed that *I*. tie. lon was significantly taller than other species (*p* < 0.05), while the remaining four species had relatively similar heights. Regarding leaf characteristics, *I*. tie. lon had larger leaf areas compared to other species. Branch diameter was the only parameter that showed no significant variation among the five species, ranging from 0.59 to 0.72 cm.

**Table 3:** Growth characteristics (mean ± SD) of five *Impatiens* species in Jiulong Mountain.

Species	Crown	Plant height	Branch diameter	Blade number	Leaf area
*I*. sui	528.99 ± 76.69b	41.46 ± 5.68b	0.59 ± 0.14a	43.76 ± 8.11a	995.00 ± 216.74a
*I*. chl	558.55 ± 254.30b	36.93 ± 9.78b	0.72 ± 0.19a	12.72 ± 3.67b	383.11 ± 143.98b
*I*. tie. lon	3354.19 ± 1559.40ab	53.38 ± 11.50a	0.59 ± 0.11a	38.70 ± 15.47ab	2067.63 ± 799.39ab
*I*. jiu	1230.80 ± 362.58a	40.05 ± 7.09b	0.64 ± 0.12a	14.06 ± 3.78b	922.36 ± 369.56ab
*I*. che	428.13 ± 151.72b	34.39 ± 9.17b	0.63 ± 0.14a	21.39 ± 8.56b	393.97 ± 162.64b

Values followed by different letters within the same column indicate significant differences among species (*P* < 0.05). Values sharing the same letter are not significantly different.

### 3.3 High-elevation endemic *Impatiens* species associate with nutrient-rich soils and distinct environmental characteristics

Significant differences in soil physicochemical properties were observed among the habitats of different *Impatiens* species (**Table 4**). For soil nutrients, the habitats of *I*. sui and *I*. tie. lon showed relatively higher levels of AK, AN, AP, and SOC compared to other species. In contrast, the habitats of *I*. che and *I*. chl generally exhibited lower soil nutrient content.

**Table 4:** Growth environment and soil physicochemical properties of five *Impatiens* species in Jiulong Mountain.

Index	*I*. sui	*I*. chl	*I*. tie. lon	*I*. jiu	*I*. che
Altitude	1029.33 ± 6.77a	531.00 ± 47.13d	981.75 ± 3.86b	855.57 ± 74.92c	727.83 ± 130.11c
Rock exposure	0.76 ± 0.07b	0.49 ± 0.20b	0.91 ± 0.01a	0.49 ± 0.24b	0.53 ± 0.36b
Litter layer	2.83 ± 0.98ab	3.08 ± 1.24a	1.75 ± 0.50b	2.57 ± 0.98ab	2.48 ± 1.03ab
Litter cover	0.80 ± 0.06a	0.58 ± 0.21ab	0.81 ± 0.03a	0.48 ± 0.28ab	0.46 ± 0.32b
Humus layer	3.00 ± 0.00a	2.58 ± 1.91a	2.50 ± 0.58a	2.29 ± 0.76a	1.49 ± 1.55a
AK	0.46 ± 0.07a	0.20 ± 0.037b	0.52 ± 0.04a	0.34 ± 0.17ab	0.20 ± 0.10b
AN	976.45 ± 147.67a	342.54 ± 62.61c	885.32 ± 149.50ab	670.25 ± 281.10b	322.57 ± 208.44c
AP	103.05 ± 32.54ab	18.17 ± 6.69c	102.48 ± 8.01a	41.79 ± 37.38bc	14.46 ± 8.45c
SOC	33.52 ± 2.90a	4.80 ± 1.14b	33.45 ± 0.94a	15.51 ± 11.61ab	5.03 ± 4.56b
pH	5.33 ± 0.38c	6.01 ± 0.44ab	5.48 ± 0.38bc	5.55 ± 0.42bc	6.32 ± 0.42a
Humidity	70.78 ± 6.65a	43.73 ± 15.23ab	62.75 ± 3.66a	60.79 ± 20.67ab	38.99 ± 11.71b
Temperature	12.87 ± 1.48d	24.08 ± 1.54b	22.10 ± 1.14c	20.94 ± 0.86c	26.47 ± 1.78a
P-Simpson	0.29.20.03b	0.68.60.12a	0.68.60.04a	0.76.70.03a	0.71.70.08a
P-Shannon	0.55.50.09c	1.54.50.31a	1.06.00.18b	1.69.60.26a	1.54.50.33a
P-Pielou	0.42.40.04c	0.71.70.09ab	0.76.70.01a	0.75.70.03a	0.68.60.05b

Values followed by different letters within the same column indicate significant differences at *p* < 0.05. Values sharing the same letter are not significantly different. P-Simpson, P-Shannon, and P-Pielou denote the Shannon-Wiener index, Simpson diversity index, and Pielou evenness index of companion plant communities, respectively.

Soil pH ranged from 5.33 to 6.32, with *I*. che habitats showing significantly higher pH than other species (*p* < 0.05) except for *I*. chl. Soil moisture content was significantly higher in the habitats of *I*. sui and *I*. tie. lon compared to *I*. che habitats (*p* < 0.05). Soil temperature decreased with increasing elevation, with the highest temperature at *I*. che habitats and the lowest at *I*. sui habitats.

Among the environmental characteristics, the habitats of *I*. sui and *I*. tie. lon showed higher rock exposure and litter coverage, while *I*. sui habitats had the thickest humus layer. Companion plant diversity indices also varied among habitats. *I*. sui showed significantly lower plant Simpson diversity, Shannon-Wiener diversity, and Pielou evenness indices compared to other species (*p* < 0.05). The plant Shannon-Wiener diversity index of *I*. tie. lon was significantly lower than all other species (*p* < 0.05) except *I*. sui. For the Pielou evenness index, *I*. che showed significantly lower values than *I*. tie. lon and *I*. jiu (*p* < 0.05), but no significant differences were found with other species.

### 3.4 Endemic Impatiens species harbor distinctive rhizosphere microbial communities

#### 3.4.1 *I*. tie. lon maintains the highest bacterial community richness despite low companion plant diversity

High-throughput sequencing generated sufficient data for all samples, with high library coverage (overag indicating adequate sequencing depth (**Supplementary Table S2**). Analysis of α-diversity indices for bacterial and fungal communities in the rhizosphere soil of five *Impatiens* species revealed significant interspecific differences (**Table 5**). For bacterial communities, *I*. tie. lon exhibited the highest community richness, with its ACE index and Chao index being significantly higher than other species (*p* < 0.001). Its microbial Shannon-Wiener diversity index also remained high, which, together with *I*. che, was slightly higher compared to the other three species. However, the microbial Simpson diversity index showed no significant differences among the five species (*p* = 0.3571).

**Table 5:** Diversity indices of rhizosphere bacterial and fungal communities in five *Impatiens* species.

	Species	M-ACE	M-Chao	M-Simpson	M-Shannon
Bacteria	*I*. sui	2644.3 ± 292.5b	2585.3 ± 267.0b	0.9977 ± 0.0005a	6.9073 ± 0.1189b
	*I*. chl	2568.6 ± 204.8b	2544.2 ± 188.2b	0.9973 ± 0.0012a	7.0255 ± 0.1201ab
	*I*. tie. lon	4020.6 ± 369.1a	3875.4 ± 356.6a	0.9964 ± 0.0038a	7.2311 ± 0.2518a
	*I*. jiu	2661.6 ± 417.8b	2603.0 ± 385.1b	0.9969 ± 0.0010a	6.8762 ± 0.1436b
	*I*. che	2959.3 ± 519.1b	2893.6 ± 480.6b	0.9978 ± 0.0005a	7.1247 ± 0.1345a
	*F* test	11.7758	10.9312	1.2269	4.7684
	*P* value	0.0004	0.0006	0.3571	0.0170
Fungi	*I*. sui	1102.4 ± 188.4b	1095.8 ± 182.6c	0.961 ± 0.0126a	4.6930 ± 0.2404a
	*I*. chl	1182.3 ± 236.9b	1176.9 ± 236.9bc	0.976 ± 0.0112a	5.1053 ± 0.4843a
	*I*. tie. lon	1659.4 ± 379.6a	1640.6 ± 375.9a	0.947 ± 0.0540a	4.9240 ± 0.5685a
	*I*. jiu	1335.5 ± 125.9ab	1328.7 ± 122.0abc	0.9707 ± 0.0168a	5.1492 ± 0.2297a
	*I*. che	1478.7 ± 219.6a	1468.4 ± 220.5ab	0.9681 ± 0.0265a	5.1528 ± 0.4470a
	*F* test	31.8887	31.4445	1.1293	3.2451
	*P* value	0.0001	0.0001	0.405	0.0534

Values followed by different letters within the same column indicate significant differences at *p* < 0.05. Values sharing the same letter are not significantly different. M-ACE, M-Chao, M-Simpson, and M-Shannon denote the ACE index, Chao index, Simpson diversity index, and Shannon-Wiener diversity index of microbial communities, respectively.

The α-diversity patterns of fungal communities showed slight differences. *I*. tie. lon and *I*. che demonstrated much higher ACE and Chao indices compared to other species, suggesting higher fungal community richness in these two species. However, the microbial Simpson and Shannon-Wiener diversity indices did not show significant differences among these five species.

#### 3.4.2 Fungal communities show greater interspecific variation than bacterial communities across *Impatiens* species

**Figure 1:**
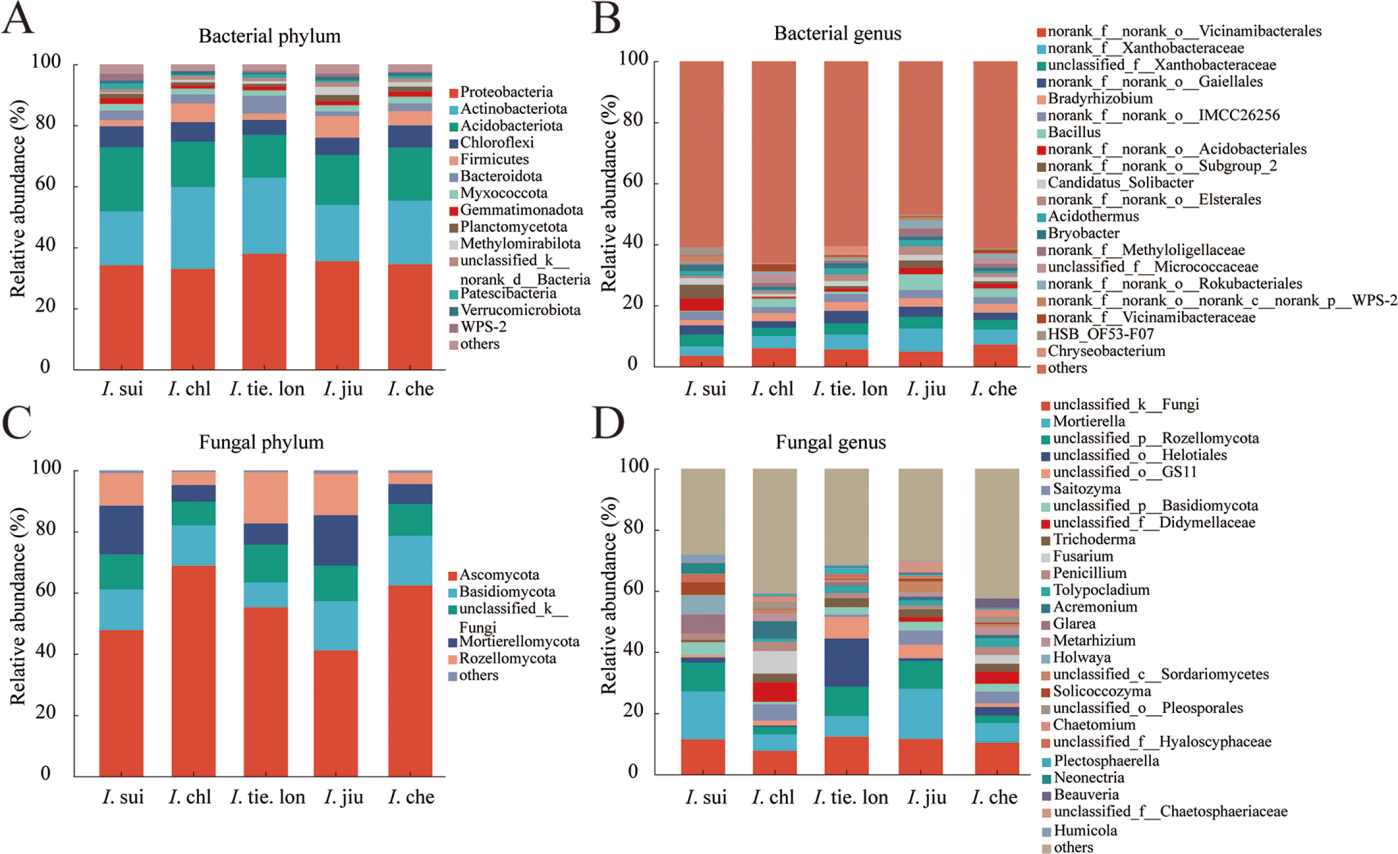
Endemic and widespread *Impatiens* species harbor distinct rhizosphere microbial community compositions, with greater interspecific variation in fungal communities. Stacked bar charts showing the relative abundance of **(A)** bacterial phyla and **(B)** bacterial genera, and **(C)** fungal phyla and **(D)** fungal genera in rhizosphere soil of five *Impatiens* species. Rhizosphere soil samples were collected from 38 quadrats across an elevation gradient (380–1050 m), with three healthy plants sampled per quadrat to create composite samples (n = 6 for *I.* sui, n = 6 for *I.* chl, n = 4 for *I.* tie. lon, n = 7 for *I.* jiu, and n = 12 for *I.* che). High-throughput sequencing targeting bacterial 16S rRNA V3-V4 regions and fungal ITS1 region was performed, followed by taxonomic annotation against Silva and UNITE databases, respectively. Note the pronounced differences in fungal community composition among species, especially for endemic *I.* sui and *I.* tie. lon.

The rhizosphere microbial communities of the five *Impatiens* species showed distinct compositional differences at both phylum and genus levels (**Figure 1**). At the bacterial phylum level, Proteobacteria dominated all samples with relative abundances of 30-35%, followed by Actinobacteriota (15-20%), Acidobacteriota and Chloroflexi (both approximately 10-15%). Although Bacteroidota, Myxococcota, and Gemmatimonadota showed relatively lower abundances, they maintained stable distributions across all samples. At the genus level, norank_f_norank_o_Vicinamibacterales was the most dominant group, accounting for over 50% in all samples. Other major genera included norank_f_Xanthobacteraceae, unclassified_f_Xanthobacteraceae, and *Bradyrhizobium*, with varying relative abundances among different *Impatiens* species. Microbial community composition analysis at the ASV level revealed that *I*. che exhibited the highest number of both bacterial and fungal ASVs, while endemic species (*I*. sui and *I*. tie. lon) showed relatively lower ASV numbers, yet each species possessed unique ASV compositions (**Supplementary Figure S1**). This species-specific pattern was also evident at higher taxonomic levels, with more pronounced differences observed in fungal communities.

Fungal community composition exhibited greater interspecific variation (**Figure 1**). At the phylum level, Ascomycota dominated all samples, but its relative abundance varied significantly among species, ranging from approximately 40% in *I*. jiu to 70% in *I*. chl. Basidiomycota, the second most dominant phylum, reached its highest relative abundance in *I*. jiu (approximately 20%). Notably, unclassified_k_Fungi was present across all samples, suggesting a considerable proportion of unidentified fungal taxa.

At the genus level (**Figure 1**), community composition showed higher heterogeneity. The distribution proportions of genera such as *Mortierella*, unclassified_p_Rozellomycota and unclassified_f_Helotiales varied significantly among different *Impatiens* species. In *I*. tie. lon samples particularly, the composition of dominant genera distinctly differed from other species, for instance, the unclassified_f_Helotiales comprised an overwhelmingly larger proportion compared to other plant species. These findings indicate that while rhizosphere microbial communities of different *Impatiens* species share certain major taxa, each species has cultivated distinctive microbial compositional characteristics, with these differences being more pronounced in fungal communities. This pattern likely reflects associations between rhizosphere microbial communities and different *Impatiens* species, possibly related to the unique rhizosphere microenvironments associated with these species during their long-term evolution.

#### 3.4.3 Endemic *I*. sui exhibits highly distinct microbial community structure separated from widespread congeners

**Figure 2:**
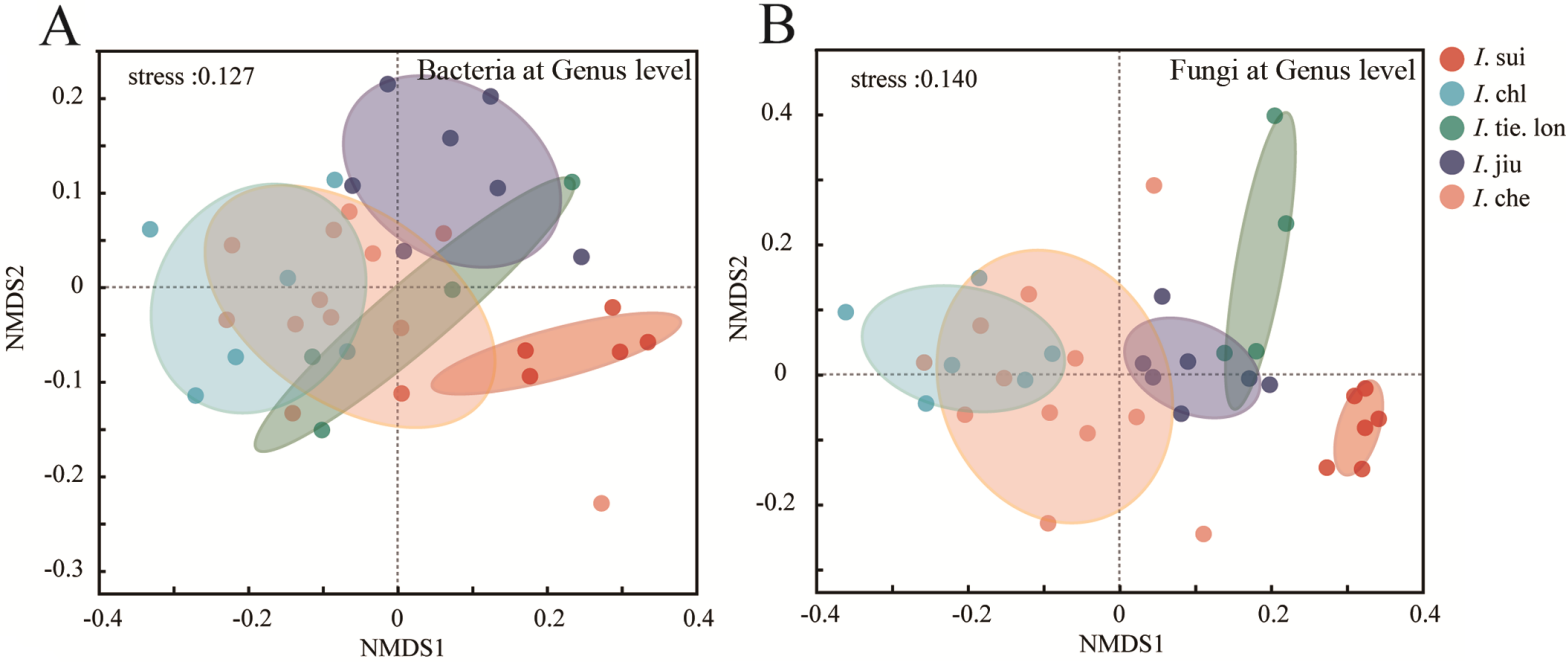
Endemic ***I.*** sui maintains highly distinct rhizosphere microbial communities separated from widespread congeners. Non-metric Multidimensional Scaling (NMDS) ordination based on Bray-Curtis distances showing **(A)** bacterial and **(B)** fungal community composition at the genus level. Samples were collected from 38 quadrats, with each point representing a composite rhizosphere soil sample from three plants. Colored ellipses indicate 95% confidence intervals for each species. Significant differences among species were confirmed by Analysis of Similarities (ANOSIM, *p* < 0.001). Note the distinct separation of endemic ***I.*** sui samples from other species in both bacterial and fungal communities, with stronger species-specific clustering observed in fungal communities (stress values < 0.2 indicate reliable ordination).

Non-metric Multidimensional Scaling (NMDS) analysis (**Figure 2**) revealed distinct species-specific distribution patterns in rhizosphere microbial communities at the genus level across the five *Impatiens* species. All NMDS analyses yielded stress values below 0.2, indicating reliable two-dimensional ordination results. Similar patterns were also observed at the ASV level (**Supplementary Figure S2**).

For bacterial communities at the genus level (**Figure 2A**), *I*. sui samples formed a tight cluster in the high positive region of NMDS1 axis, completely separated from other species, indicating unique bacterial community composition; *I*. jiu samples clustered in the positive regions of both NMDS1 and NMDS2 axes; *I*. tie. lon samples were mainly distributed in the central region; *I*. chl and *I*. che samples clustered in the negative region of NMDS1 axis, with *I*. che showing greater dispersion along NMDS2 axis.

Fungal communities exhibited stronger species differentiation (**Figure 2B**), where *I*. jiu samples were primarily distributed in the positive regions of NMDS1 axis; *I*. sui samples clustered in the positive region of NMDS1 and negative region of NMDS2, distinctly separated from other species; *I*. chl and *I*. che samples were mainly located in the negative region of NMDS1 axis but showed some dispersion along NMDS2 axis; *I*. tie. lon samples formed a relatively tight cluster, mainly in the positive regions of both axes.

Overall, ANOSIM test results indicated significant differences in rhizosphere microbial community composition among different *Impatiens* species (R = 0.756, *p* < 0.001 for bacterial communities; R = 0.823, *p* < 0.001 for fungal communities), with these differences being more pronounced in fungal communities than in bacterial communities. These distribution patterns reveal significant associations between different *Impatiens* species and their rhizosphere microbial communities, with these associations being particularly pronounced in fungal communities. Comparable patterns were observed at the ASV level (**Supplementary Figure S2**), suggesting consistency in microbial community differentiation, across different taxonomic resolutions.

### 3.5 Elevation strongly correlates with fungal community variation while multiple factors shape bacterial communities

To comprehensively understand the factors shaping rhizosphere microbial communities of different *Impatiens* species, we employed three complementary analytical approaches. Redundancy Analysis (RDA) provided a multivariate visualization of how environmental factors interact with microbial community composition, revealing that different *Impatiens* species associate with distinct environmental drivers. Spearman correlation analysis identified specific relationships between environmental factors and microbial diversity indices, uncovering particularly strong correlations between soil nutrients and fungal diversity. Mantel correlation tests examined species-specific environmental associations by analyzing correlations between distance matrices. Collectively, these approaches revealed several key patterns: (1) elevation strongly influences fungal communities, explaining 78.84% of variation compared to only 39.81% for bacterial communities; (2) soil nutrients consistently correlate with microbial diversity, particularly for fungi; (3) endemic species exhibit distinct environmental correlation patterns compared to widespread congeners; and (4) companion plant diversity positively correlates with fungal diversity.

**Figure 3:**
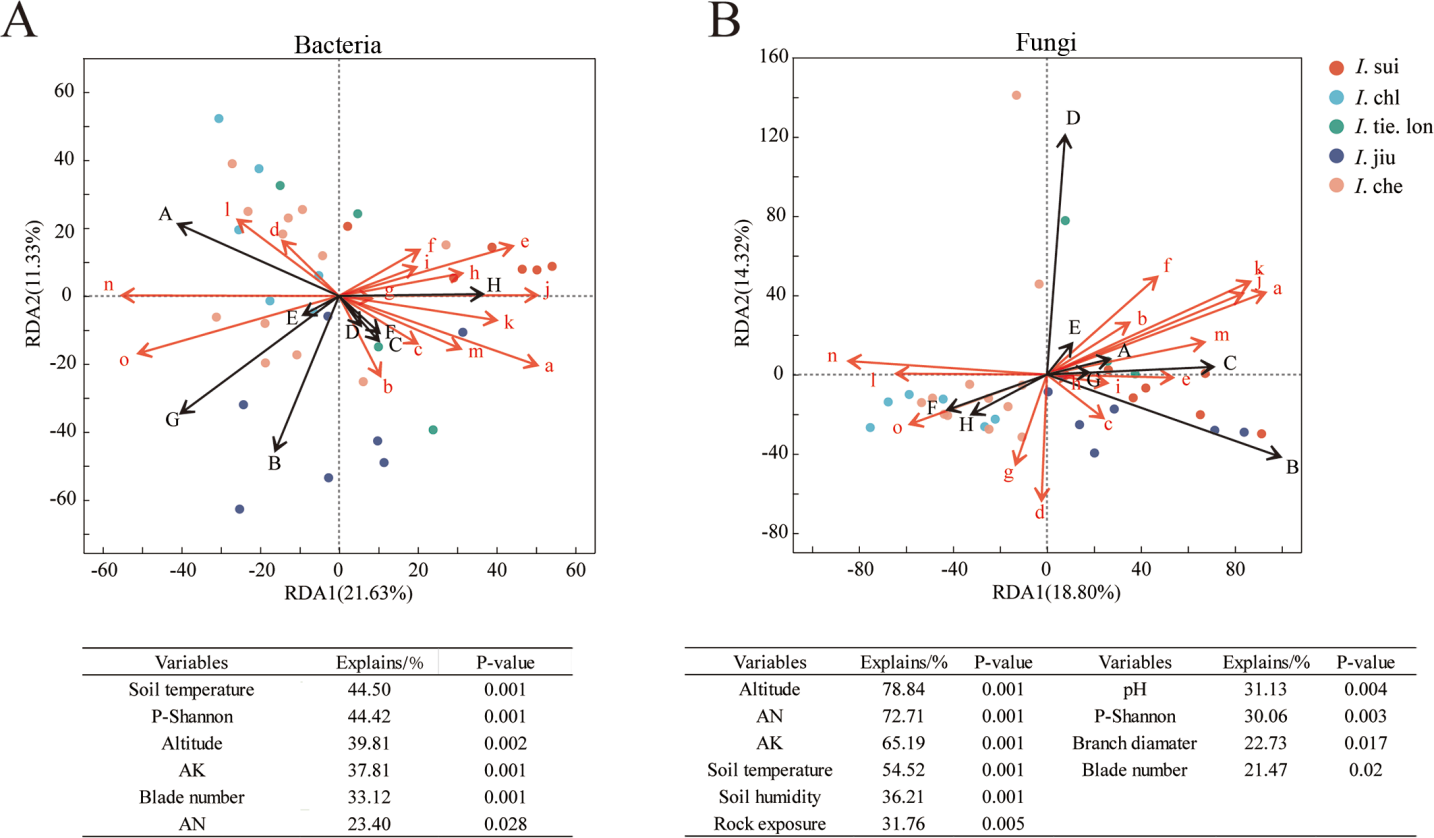
Elevation explains 78.84% of fungal community variation but only 39.81% of bacterial community variation in *Impatiens* rhizospheres. Redundancy analysis (RDA) showing relationships between environmental variables and **(A)** bacterial and **(B)** fungal genus compositions. Samples were collected from 38 quadrats across an elevation gradient (380–1050 m) with data on 15 environmental variables. Different colors represent different *Impatiens* species (red: ***I.*** sui; light blue: ***I.*** chl; dark green: ***I.*** tie. lon; dark blue: ***I.*** jiu; light red: ***I.*** che). Capital letters represent dominant microbial taxa - For bacteria **(A)**: A-Norank_f_norank_o_Vicinamibacterales, B-Norank_f_Xanthobacteraceae, C-Unclassified_f_Xanthobacteraceae, D-Norank_f_norank_o_*Gaiellales*, E-*Bradyrhizobium*, F-Norank_f_norank_o_IMCC26256, G-*Bacillus*, H-Norank_f_norank_o_Acidobacteriales. For fungi **(B)**: A-Unclassified_k_Fungi, B-*Mortierella*, C-Unclassified_p_Rozellomycota, D-Unclassified_o_Helotiales, E-Unclassified_o_GS11, F-*Saitozyma*, G-Unclassified_p_Basidiomycota, H-Unclassified_f_Didymellaceae. Red lowercase letters indicate environmental variables: a-altitude, b-crown, c-plant height, d-branch diameter, e-blade number, f-rock exposure, g-litter layer, h-litter cover, i-humus layer, j-AK (available potassium), k-AN (available nitrogen), l-pH, m-soil humidity, n-soil temperature, o-companion plant Shannon-Wiener diversity index. Tables below the plots show environmental factors with significant effects (*p* < 0.05) and their relative contributions. The significance of environmental factors’ contributions was assessed by Monte Carlo permutation tests (999 permutations). Note that ***I.*** sui samples cluster in the positive region of RDA1 axis, strongly associated with altitude and litter coverage, while soil nutrients strongly influence ***I.*** jiu distribution, and temperature and pH influence ***I.*** che and ***I.*** chl distribution.

To understand the environmental factors’ influence on rhizosphere microbial community composition, we performed Redundancy Analysis (RDA) on the rhizosphere microbial communities of five *Impatiens* species (**Figure 3**). Results indicated that both bacterial and fungal communities were significantly influenced by multiple environmental factors, with sampling points of different *Impatiens* species forming distinct segregation patterns in the RDA plots.

For bacterial communities, the first two RDA axes explained 32.96% of the total community variation (**Figure 3A**). Regarding sample distribution, *I*. jiu (dark blue) samples were mainly distributed in the negative region of RDA2 axis, closely associated with soil nutrient factors (AK, AN) and Norank_f_Xanthobacteraceae (B). *I*. sui (red) samples clustered in the positive region of RDA1 axis, strongly correlated with litter coverage (h) and Norank_f_norank_o_Acidobacteriales (H). *I*. che (light red) and *I*. chl (light blue) samples were predominantly distributed in the left half of the RDA plot, demonstrating positive correlations with soil temperature (n) and Bacillus (G). Among the environmental factors, soil temperature (44.50%) and companion plant Shannon diversity (44.42%) demonstrated the highest explanatory power, followed by altitude (39.81%) and AK (37.81%).

RDA analysis of fungal communities showed that the first two axes explained 33.12% of the variation (**Figure 3B**). Sample distribution exhibited more pronounced interspecific differences: *I*. sui samples were mainly distributed in the positive region of RDA1 axis, closely associated with altitude (a) and *Mortierella* (B); *I*. tie. lon (dark green) samples clustered in the positive region of RDA2 axis, strongly correlated with unclassified Helotiales (D); *I*. che and *I*. chl samples were distributed in the left half of the RDA plot, primarily influenced by soil moisture (m) and pH (l). Among the environmental factors, altitude emerged as the dominant driver with the highest explanatory power, followed by AN (72.71%) and AK. Soil temperature and soil moisture (36.21%) also exerted significant influences.

Overall, RDA analysis not only revealed the environmental factors’ influence on microbial communities but also illuminated habitat preferences of different *Impatiens* species. *I*. sui was predominantly found in high-altitude areas, with its rhizosphere microbial composition strongly influenced by altitude and litter factors. *I*. jiu’s distribution was closely related to soil nutrients, while *I*. che and *I*. chl were more strongly affected by temperature and pH.

Spearman correlation analysis revealed complex association patterns between rhizosphere microbial diversity and environmental factors (**Table 6**). For bacterial communities, most correlations with environmental factors were relatively weak, with only soil temperature showing a significant positive correlation with the microbial Shannon-Wiener diversity index (*p* < 0.01) and soil humidity exhibiting a significant negative correlation (*p* < 0.05). In contrast, fungal community diversity indices showed significant correlations with multiple environmental factors. The key ecological patterns included: (1) a negative relationship between elevation and fungal diversity indices, contrasting with bacterial communities which showed no significant elevation effect; (2) strong negative correlations between all soil nutrients (AK, AN, AP, SOC) and fungal diversity indices; and (3) a significant positive correlation between companion plant Shannon diversity (P-Shannon) and fungal diversity metrics (*p* < 0.01 or *p* < 0.05), suggesting that more diverse plant communities support higher fungal diversity in the rhizosphere.

**Table 6:** Spearman correlation analysis between rhizosphere microbial diversity indices and environmental factors in five *Impatiens* species.

Index	M-ACE		M-Chao		M-Simpson		M-Shannon	
	Bacteria	Fungi	Bacteria	Fungi	Bacteria	Fungi	Bacteria	Fungi
Altitude	0.173	-0.055	0.171	-0.052	0.018	-0.441^**^	-0.191	-0.386^*^
Crown	0.227	0.062	0.236	0.056	-0.130	-0.046	-0.078	-0.053
Plant height	0.207	-0.020	0.210	-0.024	-0.201	0.224	-0.065	0.209
Branch diameter	0.042	0.332	0.039	0.332	-0.284	0.389^*^	-0.016	0.429^*^
Blade number	0.299	-0.280	0.290	-0.280	0.153	-0.169	0.101	-0.199
Leaf area	-0.013	0.086	-0.001	0.080	-0.226	-0.120	-0.252	-0.153
Rock exposure	0.238	-0.267	0.232	-0.264	-0.015	-0.367^*^	0.059	-0.370^*^
Litter layer	-0.008	0.233	-0.005	0.227	-0.028	0.265	-0.068	0.249
Litter cover	0.164	-0.210	0.161	-0.210	0.082	0.014	-0.013	-0.013
Humus layer	0.046	-0.154	0.045	-0.153	0.068	-0.116	-0.116	-0.150
AK	0.064	-0.608^**^	0.064	-0.609^**^	0.172	-0.500^**^	-0.125	-0.623^**^
AN	-0.018	-0.496^**^	-0.015	-0.499^**^	0.047	-0.391^*^	-0.293	-0.504^**^
AP	0.096	-0.392^*^	0.096	-0.394^*^	0.063	-0.416^*^	-0.187	-0.469^**^
SOC	0.033	-0.422^*^	0.035	-0.424^*^	0.036	-0.363^*^	-0.242	-0.449^**^
pH	-0.041	0.244	-0.042	0.247	0.003	0.255	0.220	0.311
Soil humidity	-0.018	-0.351^*^	-0.014	-0.355^*^	-0.150	-0.343^*^	-0.360^*^	-0.445^**^
Soil temperature	0.119	0.440^**^	0.116	0.444^**^	0.185	0.215	0.481^**^	0.367^*^
P-Simpson	-0.119	0.298	-0.110	0.297	-0.064	0.138	0.068	0.176
P-Shannon	-0.196	0.442^**^	-0.189	0.441^**^	-0.080	0.347^*^	0.034	0.390^*^
P-Pielou	0.125	0.219	0.132	0.219	-0.038	-0.021	0.188	0.062

The table shows Spearman correlation coefficients between microbial diversity indices (M-ACE; M-Chao; M-Simpson, microbial Simpson diversity index; M-Shannon, microbial Shannon-Wiener diversity index) and environmental factors. Asterisks (*) and (**) indicate significant correlations at *p* < 0.05 and *p* < 0.01, respectively. Negative values indicate negative correlations and positive values indicate positive correlations. Environmental factors include: altitude, plant traits, habitat characteristics, soil properties, and companion plant diversity indices (P-Simpson, Simpson diversity index; P-Shannon, Shannon-Wiener diversity index; P-Pielou, Pielou evenness index). AK-Available potassium, AN-available nitrogen, AP-available phosphorus, SOC-soil organic carbon.

**Figure 4:**
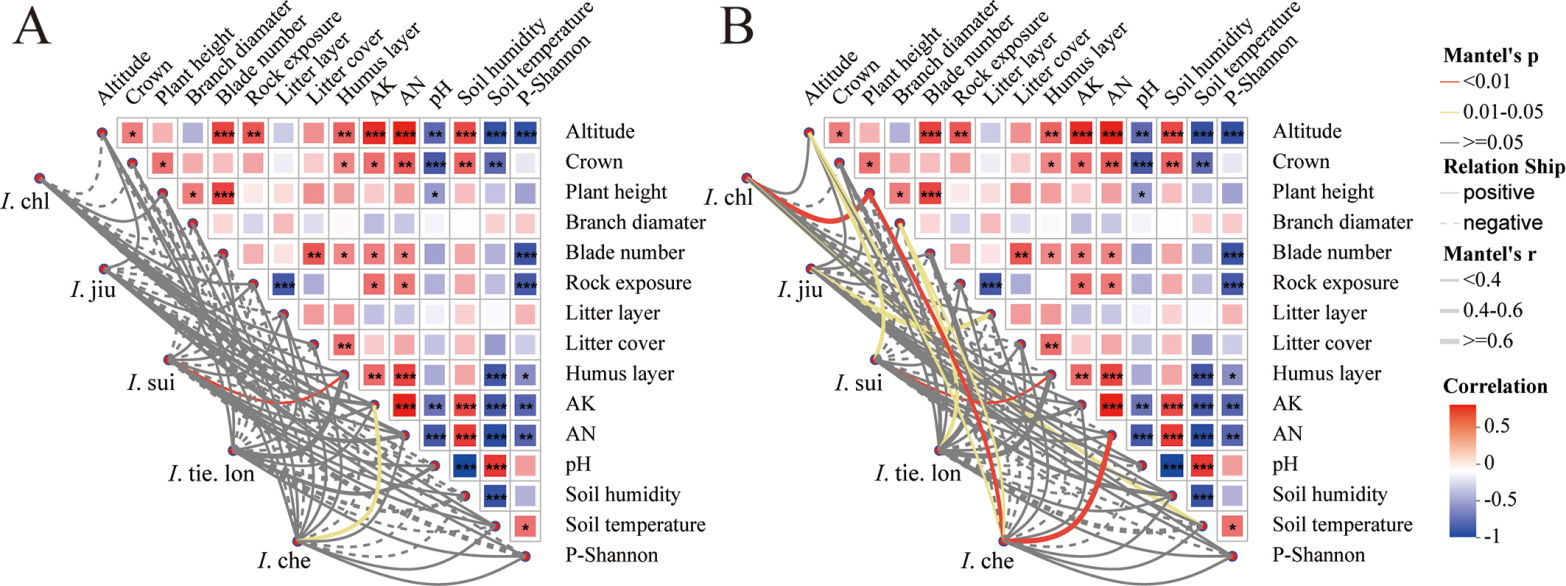
Different *Impatiens* species show distinct correlation patterns with environmental factors, with stronger fungal response to these variables. Mantel correlation network analysis between environmental factors and rhizosphere **(A)** bacterial and **(B)** fungal communities at genus level. Data from 38 quadrats were analyzed, with each quadrat representing one of five *Impatiens* species across an elevation gradient. In the heatmap, colors indicate the strength and direction of Mantel correlation coefficients: red represents positive correlations (0-0.5) and blue represents negative correlations (-1 to -0.5), with significance levels indicated by asterisks (*p < 0.05, **p < 0.01, ***p < 0.001). Network diagrams show correlations between each *Impatiens* species and environmental factors: red lines indicate highly significant correlations (*p* < 0.01), yellow lines indicate significant correlations (*p* < 0.05), and grey lines indicate non-significant correlations (*p* ≥ 0.05); solid lines represent positive correlations and dashed lines represent negative correlations; line thickness is proportional to the absolute correlation coefficient value (|r|). Environmental factors include: plant growth traits (crown, plant height, branch diameter, blade number), soil properties (pH, AK (available potassium), AN (available nitrogen)), environmental characteristics (altitude, rock exposure, litter layer thickness, litter cover, humus layer thickness, soil humidity, soil temperature), and companion plant diversity index (P-Shannon, Shannon-Wiener diversity index). Note that soil nutrients (AK, AN), pH, and altitude play central roles in correlation networks, with endemic ***I.*** sui showing significant positive correlations with humus layer thickness and altitude, while fungal communities demonstrate more complex and stronger correlations with environmental variables than bacterial communities.

Mantel correlation analysis revealed distinct environmental association patterns among the five *Impatiens* species (**Figure 4**). The most ecologically significant patterns emerged in species-specific environmental correlations. Endemic *I*. sui showed strong positive correlations with humus layer thickness and altitude, characteristic of its restricted distribution at high elevations with well-developed organic soil layers. In contrast, the widespread *I*. che exhibited positive correlations with available nitrogen, highlighting different resource requirements. Notably, fungal communities showed more complex and stronger correlations with environmental variables than bacterial communities, reinforcing our RDA results.

These species-specific correlation patterns suggest that different *Impatiens* species have developed unique associations with soil environmental factors, likely representing their distinct ecological niches and adaptation strategies. However, our observational approach cannot definitively determine the direction of causality in these complex and interconnected relationships among plants, environmental factors, and microbial communities.

## 4 Discussion

### 4.1 Endemic *Impatiens* species maintain unique rhizosphere microbiomes with stronger differentiation in fungal communities

Our study revealed significant interspecific differences in rhizosphere microbial communities among five *Impatiens* species, with these distinctions being more pronounced in fungal communities. *I.* tie. lon exhibited the highest bacterial community richness, while *I.* che and *I.* tie. lon showed higher fungal community richness. This pattern aligns with plant species-specific rhizosphere effects documented by Fitzpatrick et al. (2018), who found that plant phylogenetic relationships explained approximately 17% of rhizosphere bacterial community variation.

NMDS analysis showed that *I.* sui’s rhizosphere microbial community was significantly separated from other species, likely related to its specific habitat requirements. This separation may partially explain why the *I.* sui was endemic to Jiulong Mountain. Proteobacteria showed significant differences in relative abundance among different species, which is noteworthy given their crucial roles in plant nutrient acquisition and stress response (Delgado-Baquerizo et al., 2018). For fungal communities, we observed significant differences in Ascomycota and Basidiomycota distribution, suggesting species-specific fungal associations. Notably, unclassified_f_Helotiales showed unique distribution patterns in *I.* tie. lon samples, possibly associated with this species’ larger crown at high elevation. Recent studies by Bahram et al. (2018) demonstrate that fungal community assembly at local scales is influenced by host identity and functional traits.

From an evolutionary perspective, these interspecific differences might represent important features developed during *Impatiens* adaptive radiation. For endemic species like *I.* sui and *I.* tie. lon, their unique rhizosphere microbial composition might constitute a crucial component of local adaptation. Magotra et al. (2024) suggest that plant-associated microbiomes can facilitate rapid adaptation to new environments by extending host phenotypic plasticity. Despite these interspecific differences, we identified shared ASVs across species, suggesting the presence of core microbial groups that might serve fundamental roles in *Impatiens* development, which aligns with previous findings of conserved core root microbiomes (Yeoh et al., 2017).

While our results demonstrate clear associations between specific *Impatiens* species and distinct microbial communities, the observational nature of our study limits our ability to definitively determine causality in these relationships. Several alternative interpretations warrant consideration: (1) the plant species may directly shape their rhizosphere communities through species-specific root exudates, as suggested by Zhalnina et al. (2018); (2) both plants and microbes may independently respond to the same environmental gradients, resulting in parallel distribution patterns; or (3) historical contingency might play a role, with initial colonization patterns persisting through ecological drift. The strong correlations between environmental factors and microbial communities suggest that at least some of the observed species-specific patterns may reflect shared environmental preferences rather than direct plant selection. Future experimental approaches, such as reciprocal transplants or common garden experiments, would provide crucial insights needed to disentangle these possibilities.

### 4.2 Elevation exerts stronger influence on fungal than bacterial community variation in *Impatiens* rhizospheres

Environmental factors significantly correlated with *Impatiens* rhizosphere microbial community structure. The elevational gradient explained 78.84% of fungal community variation but only 39.81% of bacterial variation—a striking difference in response sensitivity. This differential response aligns with findings by Nottingham et al. (2018) and recent meta-analyses showing fungi typically exhibit stronger biogeographic patterns than bacteria due to their larger cell sizes, lower dispersal rates, and more specialized ecological niches (Chu et al., 2020).

Our study employed multiple analytical approaches to thoroughly investigate the environmental factors shaping rhizosphere microbial communities. Each analytical method provided unique insights: RDA allowed visualization of multivariate relationships between environmental factors and community composition, revealing that endemic *I*. sui’s microbial community correlated strongly with elevation and litter factors. Spearman correlation analysis identified specific relationships between environmental factors and diversity indices, highlighting the stronger correlations with fungal than bacterial diversity. Mantel tests complemented these approaches by examining species-specific environmental associations through distance matrices, demonstrating that endemic and widespread *Impatiens* species respond differently to the same environmental gradients. The consistency of patterns across these different analytical approaches substantially enhances our confidence in the ecological relationships observed, particularly the differential responses of fungal and bacterial communities to elevation gradients. This methodological triangulation approach is increasingly recognized as valuable in microbial ecology studies (Delgado-Baquerizo et al., 2016), providing far more convincing evidence than any single analytical method alone.

Our analysis further revealed contrasting diversity patterns along the elevation gradient. Fungal diversity indices (i.e., microbial Simpson and Shannon-Wiener diversity indices) were significantly negatively correlated with altitude, while bacterial diversity showed no significant relationship. This pattern of declining fungal diversity with increasing elevation aligns with several previous studies (Delgado-Baquerizo et al., 2016; Nottingham et al., 2018). However, the relationship between elevation and microbial diversity appears to be ecosystem-dependent, as Chu et al. (2020) found no consistent negative association between elevation and microbial diversity across different ecosystems. This suggests that elevation effects on microbial communities are likely mediated by local environmental conditions particular to subtropical mountain forests.

Soil nutrients (AK, AN, and AP) emerged as key factors correlated with microbial community structure. Higher-elevation habitats of *I.* sui and *I.* tie. lon exhibited both higher soil nutrient content and distinct microbial communities. This pattern likely reflects plant-soil-microbe interactions, as nutrients both directly affect microbial growth and indirectly influence communities by altering plant root exudates (Zhalnina et al., 2018). We also found a significant positive correlation between soil temperature and bacterial Shannon-Wiener diversity index, which supports previous findings that temperature serves as a key driver of microbial diversity and metabolic activity in soil ecosystems (Zhou et al., 2016).

Different *Impatiens* species showed unique patterns of environmental correlations. *I.* sui’s microbial community primarily correlated with elevation and humus layer thickness, while *I.* jiu and *I.* che communities associated with litter layer thickness and available nitrogen, respectively. These differential responses likely reflect species-specific ecological adaptations (Singh et al., 2020). A key unresolved question is whether *Impatiens* species have determined their ecological niches or whether these niches have shaped the evolutionary adaptation of the plants. This classic ecological question of niche construction versus environmental filtering (Odling-Smee et al., 2013) cannot be resolved through observational studies alone, but our findings establish a valuable foundation for future experimental work to address these fundamental questions.

### 4.3 Plant size positively correlates with microbial diversity with species-specific associations between plant traits and key microbial taxa

Plant growth parameters showed complex associations with rhizosphere microbial communities. *I.* tie. lon, with the largest crown area, height and leaf area, maintained the highest bacterial diversity and fungal richness. This positive correlation between plant size and microbial diversity corresponds with previous findings that larger plants support more diverse microbial communities through increased root exudation (Eisenhauer et al., 2017).

Species-specific trait-microbe correlations were evident, including associations between *I.* sui’s growth parameters and *Mortierella* abundance, and between *I.* chl’s traits and *Saitozyma* distribution. These patterns suggest niche-specific adaptations in plant-microbe interactions. Recent research demonstrates that plant functional traits often more accurately predict fungal community composition than phylogenetic relationships alone (Bahram et al., 2018).

### 4.4 Companion plant diversity enhances fungal diversity despite endemic *I*. sui maintaining unique microbiomes with low companion diversity

Companion plant diversity significantly correlated with *Impatiens* rhizosphere microbial communities, explaining 30%-45% of community variation. We found positive correlations between companion plant diversity (Shannon-Wiener diversity index) and fungal diversity indices, consistent with Lepinay et al. (2024) finding that high-diversity plant communities harbored a higher diversity of fungi in a Central European grassland. This relationship likely stems from increased resource heterogeneity in diverse plant communities, as Eisenhauer et al. (2017) demonstrated that higher plant diversity increases microbial biomass through enhanced root exudation. Additionally, Chu et al. (2020) demonstrated that plant diversity influences microbial network structure and keystone taxa.

However, this relationship is not always straightforward. Schlatter et al. (2015) found plant community richness could sometimes negatively affect bacterial diversity, suggesting complex above-below-ground interactions. The functional significance of these plant-microbe associations has been highlighted in recent studies. For example, Li et al. (2024) demonstrated that companion planting of white clover (*Trifolium repens*) with orchard grass (*Dactylis glomerata*) enhanced gene functions related to nitrogen and carbon fixation in rhizosphere soil, confirming that plant community composition can directly shape microbial functional potential.

Interestingly, *I.* sui, despite having significantly lower companion species diversity, maintained a unique rhizosphere microbial community. This pattern is likely attributable to specific plant and soil properties characteristic of *I*. sui habitats (Grayston et al., 1998; Garbeva et al., 2004). Companion plants may also influence microbial communities through litter quality and quantity variations, as Urbanová et al. (2015) found strong associations between forest litter diversity and soil fungal composition. The distinct microbial communities across *Impatiens* species indicate that understory herbs maintain specific rhizosphere assemblages despite the broader influence of forest vegetation.

### 4.5 Conservation strategies for endemic *Impatiens* should protect both plants and their specialized rhizosphere microbiomes

Of particular conservation concern is *I.* sui, which is restricted to a single distribution site within Jiulong Mountain. This extreme geographic restriction, combined with its distinct rhizosphere microbiome, underscores its exceptional vulnerability to extinction. The loss of this single population would represent the global extinction of this species and its associated microbiome, highlighting the urgent conservation value of our findings.

Our results suggest effective conservation must protect both plant species and their associated microbiomes, which could be supported by recent conservation biology frameworks (Guerra et al., 2022). The strong correlation between environmental factors and microbial communities indicates habitat protection is crucial. The impact of companion plant diversity on rhizosphere microbiomes emphasizes preserving entire plant communities rather than individual species alone. Bahram et al. (2018) suggest that maintaining diverse plant communities enhances microbial diversity and functional redundancy, which may increase ecosystem resilience to disturbances and environmental changes.

We propose three key conservation recommendations: (1) Establish buffer zones around endemic *Impatiens* populations to protect soil environments; (2) Implement systematic monitoring of both plant populations and their rhizosphere microbial communities; and (3) When *ex-situ* conservation is necessary, transplant original habitat soil to maintain essential microbial associations.

### 4.6 Experimental approaches needed to establish causality in plant-microbe relationships beyond observational correlations

A key limitation of our study is its observational nature, which allows us to identify correlative relationships but not establish causality. While our multifactorial analyses attempted to partition environmental and plant trait contributions, natural co-variation in field settings limits our ability to definitively attribute microbiome differences to plant species effects alone. The strong correlations we observed between environmental factors, plant species, and rhizosphere microbiomes could result from multiple underlying processes, including: (1) direct selection of microbes by plants, (2) independent responses of both plants and microbes to environmental gradients, or (3) complex feedback loops where plants modify soil properties that subsequently affect microbial communities.

Ideally, reciprocal transplant experiments or common garden studies would address species-specific effects (Li et al., 2024; Tao et al., 2024; Dostál, 2025), but such manipulations present significant challenges when working with endemic species having specific habitat requirements and conservation restrictions. Recent methodological advances proposed by Halbritter et al. (2020) for standardized field experiments could be adapted for future work with these sensitive species. Nevertheless, our approach provides valuable baseline data on rhizosphere microbiomes as they exist in natural habitats.

Future research could include greenhouse experiments with cultivable *Impatiens* species, microcosm studies examining specific plant-microbe interactions, metagenomic approaches to assess functional potential, and longitudinal studies tracking plant-microbe dynamics in response to environmental changes. Additionally, functional analysis of microbiomes through metagenomics or metatranscriptomics would shed light on whether the observed taxonomic differences translate to functional differences relevant to plant ecology. Controlled experiments systematically manipulating either plant species composition or environmental conditions would be essential for unraveling the complex relationships we observed in this study.

## 5 Conclusion

Our study revealed distinct rhizosphere microbial communities among five *Impatiens* species in subtropical mountain ecosystems, with endemic species harboring unique microbiomes. Fungal communities displayed stronger species-specificity than bacterial communities, most notably in endemic *I*. sui. Environmental factors, especially elevation, showed stronger correlations with fungal (78.84%) than bacterial (39.81%) community variation, pointing to distinct assembly mechanisms for these microbial groups.

Soil nutrients, pH, and plant traits exhibited species-specific correlations with microbial communities. *I*. tie. lon’s larger crown and height were associated with higher bacterial diversity and fungal richness, while companion plant diversity positively correlated with fungal diversity indices. *I*. sui, restricted to a single distribution site, faces exceptional extinction vulnerability—the loss of this population would result in the global extinction of both the species and its unique microbiome.

These findings enhance our understanding of plant-soil-microbe associations in endemic species and provide valuable insights for *Impatiens* conservation. Our results emphasize the importance of considering both above- and below-ground components in conservation strategies, especially for endemic species with specialized rhizosphere communities. Future research should investigate the functional implications of these species-specific microbiomes and their potential contributions to plant adaptation to local environments through controlled experiments.

## Data Availability

All relevant data is contained within the article: The original contributions presented in the study are included in the article/**Supplementary Material**, further inquiries can be directed to the corresponding author.
