# Supplementary material for: Interspecific variation and environmental drivers of rhizosphere microbiomes in endemic Impatiens species
Source: Front Plant Sci. 2025 May 8;16:1567041. doi: 10.3389/fpls.2025.1567041 (PMC12095246; doi:10.3389/fpls.2025.1567041)
Supplement: Supplementary file 1 [file DataSheet1.pdf]

Interspecific variation and environmental drivers of rhizosphere microbiomes in endemic *Impatiens* species: evidence from subtropical mountains of China

Zhansheng Tang[1,2†], Lina Xie[1†], Junjie Chen[1], Yunquan Wang[3], Yalei Li[1*]

[1]College of Ecology, Lishui University, Lishui, Zhejiang, China

[2]The Administration Center of Jiulong Mountain National Nature Reserve, Lishui, Zhejiang, China

[3]College of Life Sciences, Zhejiang Normal University, Jinhua, Zhejiang, China

*** Correspondence:**
Yalei Li (Y.-L. Li)
liyalei@lsu.edu.cn
† These authors contributed equally

Supplementary Material

1. Supplementary Table S1

TABLE S1. Parameters measured in all quadrats for each *Impatiens* species

Parameter category	Parameters measured	Measured in all quadrats?
Environmental factors	Elevation (m)	Yes
	Slope gradient	Yes
	Slope aspect	Yes
	Slope position (upper, middle, lower)	Yes
	Rock exposure (%)	Yes
	Litter layer thickness (cm)	Yes
	Litter cover (%)	Yes
	Humus layer thickness (cm)	Yes
Plant traits	Crown area (cm^2)	Yes
	Plant height (cm)	Yes
	Branch diameter (cm)	Yes
	Blade number	Yes
	Leaf area (cm^2)	Yes
Soil properties	Available potassium (AK) ($mg \cdot kg^{-1}$)	Yes
	Available nitrogen (AN) ($mg \cdot kg^{-1}$)	Yes
	Available phosphorus (AP) ($mg \cdot kg^{-1}$)	Yes
	Soil organic carbon (SOC) (%)	Yes
	Soil pH	Yes

	Soil moisture content (%)	Yes
	Soil temperature (°C)	Yes
Companion plant diversity	Shannon-Wiener diversity index (H')	Yes
	Simpson diversity index (D)	Yes
	Pielou evenness index (J)	Yes
Rhizosphere soil collection	Rhizosphere soil samples	3 individuals per quadrat

2. Supplementary Table S2

Table S2 presents the raw sequence counts, effective sequence counts, and library coverage for bacterial and fungal sequencing from the rhizosphere soil of five Impatiens species (Table S1). Overall, I. tie. lon showed the highest number of raw and effective sequences for both bacteria and fungi, suggesting potentially higher microbial diversity or abundance in its rhizosphere. The coverage rates remained high (≥ 0.97) across all samples, indicating sufficient sequencing depth and minimized bias from undersampling.

TABLE S2. Sequencing information of rhizosphere bacterial and fungal communities in five *Impatiens* species

Species	Raw sequences		Effective sequences		Library coverage	
	Bacteria	Fungi	Bacteria	Fungi	Bacteria	Fungi
I. sui	76859±19603b	75888±26810bc	44852±11783b	73065±25411bc	0.9918±0.0057a	0.9989±0.0013a
I. chl	59837±3027c	66026±5859c	34192±2069c	63565±5645c	0.9975±0.0023a	0.9994±0.0003a
I. tie. lon	112265±3391a	144522±50965a	63453±2416a	138272±49509a	0.9774±0.0029b	0.9958±0.0020b
I. jiu	74071±15753bc	68058±6959c	42045±9492bc	64928±6507c	0.9926±0.0056a	0.9991±0.0005a
I. che	72370±15375bc	88160±9406b	41625±8701bc	84788±9222b	0.9916±0.0064a	0.9979±0.0007a

Note: Raw sequences, effective sequences, and library coverage are key indicators for quality assessment of soil 16S rRNA and ITS sequencing.

3. Supplementary Figure S1

Microbial community structure analysis revealed both species-specific patterns and shared characteristics in bacterial and fungal community compositions across the rhizosphere soil of five *Impatiens* species (Figure S1).

At the ASV level of bacterial communities, *I.* che exhibited the highest number of ASVs, obviously exceeding other species, followed by *I.* jiu, while *I.* sui, *I.* chl, and *I.* tie. lon showed relatively lower ASV numbers (Figure S1). Venn diagram analysis indicated that while these five species shared a certain number of ASVs in their rhizosphere soil, each species also possessed its unique ASV composition, suggesting that different *Impatiens* species might selectively cultivate specific rhizosphere bacterial groups. At the genus level, the pattern of inter-species differences was similar to that at the ASV level. *I.* che maintained the highest genus-level diversity (1,048 genera), which was much higher than the other species. Notably, despite differences in total genus numbers, the proportion of shared core genera among species was higher compared to the ASV level, indicating convergence in microbial composition at higher taxonomic levels.

Fungal community analysis demonstrated patterns similar to bacterial communities, but with more pronounced inter-species differences (Figure S1). At the ASV level, *I.* che contained markedly higher fungal ASVs (12,153), approximately 1.8 times that of the second-highest species *I.* jiu (6,669). *I.* sui, *I.* chl, and *I.* tie. lon showed relatively lower ASV numbers at 4,230, 5,281, and 4,723, respectively. At the genus level, *I.* che similarly maintained the highest diversity (1,036 genera), while other species ranged between 614-725 genera.

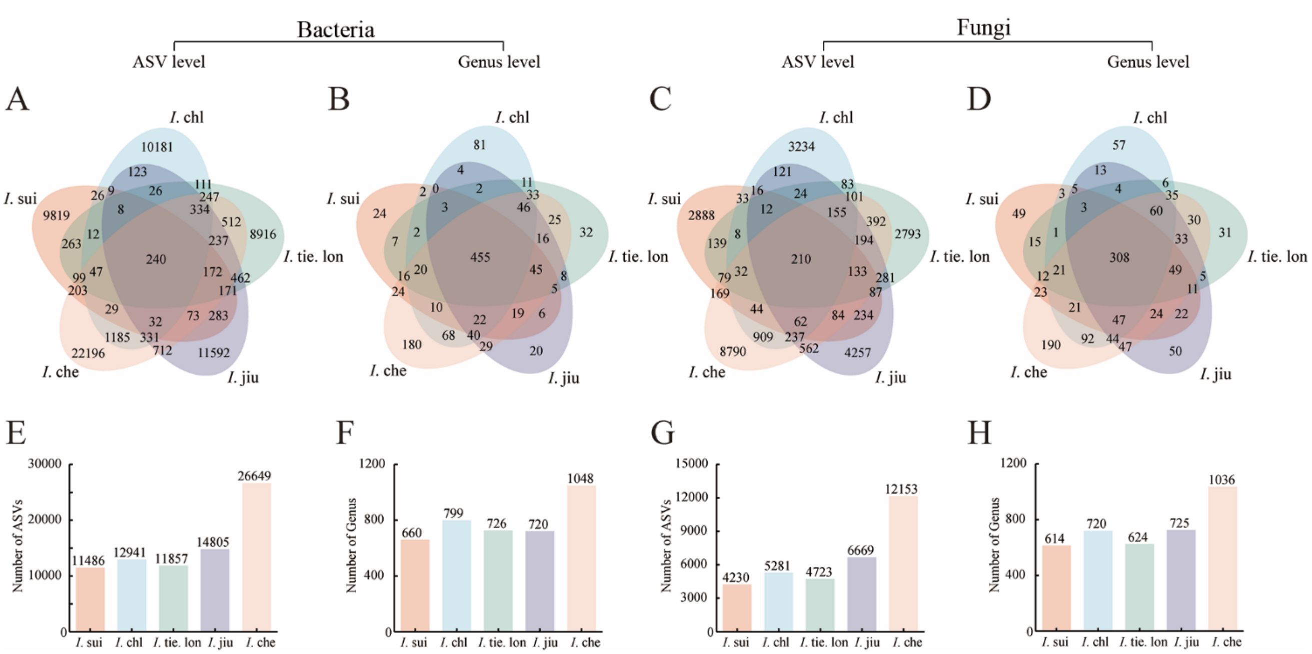

FIGURE S1 Venn diagrams and bar charts of ASV (Amplicon Sequence Variant) and genus-level distribution in the rhizosphere microbial communities of five *Impatiens* species.

4. Supplementary Figure S2

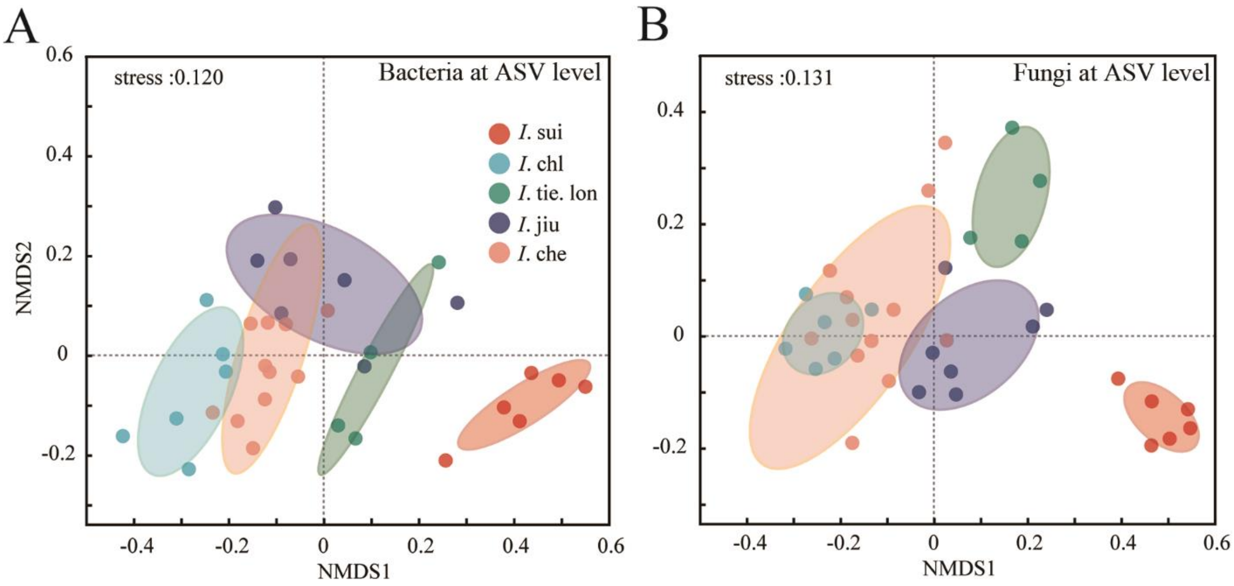

FIGURE S2 Non-metric Multidimensional Scaling (NMDS) analysis of microbial communities at the

ASV level in the rhizosphere soil of *Impatiens* species. (A) Bacterial communities and (B) fungal communities at ASV level. Each point represents an individual soil sample, and colored ellipses indicate the approximate distribution range of samples for each species (*I*. sui, *I*. chl, *I*. tie. lon, *I*. jiu, and *I*. che). Stress values denote the goodness of fit of NMDS analysis.
